# Loss of Schlafen3 influences the expression levels of Schlafen family members in ileum, thymus, and spleen tissue

**DOI:** 10.7717/peerj.8461

**Published:** 2020-01-28

**Authors:** Emilie E. Vomhof-DeKrey, Josey Umthun, Marc D. Basson

**Affiliations:** Departments of Surgery, Pathology, and Biomedical Sciences, University of North Dakota, Grand Forks, ND, USA

**Keywords:** Regulatory feedback loop, Small intestine, Mucosa, Immune cells

## Abstract

**Background:**

The Schlafen (Slfn) family proteins are important for regulation of cell growth, cell differentiation and cell cycle progression. We sought to distinguish Slfn family expression in Slfn3 knockout (KO) mice after RNA sequencing analysis of Slfn3KO vs. wildtype (WT) mice revealed varying expressions of Slfn family in ileal mucosa.

**Methods:**

Quantitative PCR analysis of Slfn members was evaluated in ileal mucosa, thymus and spleen tissue since Slfn family members have roles in differentiating intestinal and immune cells.

**Results:**

Ileal mucosa of Slfn3KO mice displayed a decrease in Slfn3, 4, 8 and 9 while Slfn1 and 5 increased in mRNA expression vs. WT mice. Thymic tissue had a Slfn9 increase and a Slfn4 decrease while splenic tissue had a Slfn8 and Slfn9 increase in Slfn3KO mice vs. WT mice. These differential expressions of Slfn members could indicate a feedback regulatory mechanism within the Slfn family. Indeed, MATCH^™^ tool from geneXplain predicted that all Slfn members have regions in their promoters for the Kruppel-like factor-6 transcription factor. In addition, NFAT related factors, ING4, ZNF333 and KLF4 are also predicted to bind in up to 6 of the 8 Slfn promoters. This study further describes a possible autoregulatory mechanism amongst the Slfn family members which could be important in how they regulate the differentiation of various cell types.

## Introduction

The Schlafen (Slfn) protein family was first identified by Schwarz, Katayama & Hedrick (1998) in thymocytes. They originally identified, mouse Slfn1, Slfn2, Slfn3 and Slfn4. These genes were expressed differentially during thymocyte maturation and T cell activation. The most prominent trait of this gene family is their effect on cell growth, cell differentiation, and progression through the cell cycle (Schwarz, Katayama & Hedrick, 1998). Slfn1 and Slfn2 are in Group I and are the shortest Slfns (37–42 kDa) (Mavrommatis, Fish & Platanias, 2013). Slfn1 induction has been found to arrest thymocytes at the G_0_/G_1_ stage of the cell cycle (Schwarz, Katayama & Hedrick, 1998) while a Slfn2 mutation (*elektra* mutation) causes activated T cells to undergo apoptosis via the intrinsic pathway. This increased the *elektra* mice to be more susceptible to bacterial and viral infections (Berger et al., 2010).

Group II Slfns include Slfn3 and Slfn4 and are intermediate in length (58–68 kDa). Slfn3 is upregulated during CD4^+^ CD25^−^ T effector cell activation but downregulated upon activation and proliferation of CD4^+^ CD25^+^ T regulatory cells (Condamine et al., 2010). The role of Slfn4 has been demonstrated to restrain the maturation of T cells and the differentiation of macrophages (Liu et al., 2018; Van Zuylen et al., 2011). Additionally, Slfn3 is important in the regulation of intestinal differentiation, development and maturation (Kovalenko & Basson, 2013; Kovalenko et al., 2013; Patel et al., 2009; Walsh et al., 2012; Yuan et al., 2010). The expression of Slfn3 in intestinal epithelial cells correlates directly with the expression of the epithelial differentiation markers sucrase isomaltase, villin1 and dipeptidyl peptidase 4, and this depends upon the Slfn3 P-loop region of its N-terminus (Chaturvedi et al., 2014; Kovalenko et al., 2013; Yuan et al., 2010).

Finally, the Group III Slfns, consisting of Slfn5, 8, 9, 10-pseudogene and 14, are the largest Slfns (100–104 kDa). In comparison to the Group I and II Slfns that are localized to the cytoplasm, the Group III Slfns contain a nuclear localization signal in the C-terminus and have been shown to be co-localized with the phosphorylated RNA polymerase II and SC-35 within the nucleus (Neumann et al., 2008). Slfn5 and Slfn8 have also been demonstrated to be involved in the modulation of T cells by inhibiting their proliferation and regulating the T cell activation (Geserick et al., 2004; Liu et al., 2018).

In a previous study, we investigated the role of Slfn3 in intestinal differentiation and maturation through the use of Slfn3 knockout (Slfn3KO) mice. We determined that Slfn3KO mice gain less weight in comparison to wild type (WT) littermates when pair-fed and that this is most striking in the female Slfn3KO mice (Vomhof-DeKrey et al., 2019). We have previously assessed the genome RNA expression in the intestinal mucosa by Illumina RNA sequencing and quantitative PCR (qPCR) and determined that Slfn3KO mice have sex-dependent disturbances in metabolic pathways, adipogenic and ketogenic genes, intestinal differentiation markers, glucose transporter 2, sodium glucose transporter 1, Notch2 and Caudal Type Homeobox 2 (Cdx2) (Vomhof-DeKrey et al., 2019). Furthermore, the RNA sequencing indicated decreased expression of immune system pathways for IgA production, leukocyte transendothelial migration and B cell receptor signaling (Vomhof-DeKrey et al., 2019). Therefore, since the intestinal mucosa is a heterogenous environment of intestinal epithelial cells and immune cells and as Slfn family members have been characterized to play significant roles in intestinal epithelial cell and T cell maturation, differentiation, and activation as summarized above, we sought to determine if the loss of Slfn3 affected Slfn family gene expression within the ileal mucosa and also studied the thymus and spleen for comparison as exemplars of the immune system.

## Methods

### Mice

Slfn3KO mice were obtained from Dr. Akira at Osaka University, Japan, and were studied under a University of North Dakota Institutional Animal Care and Use Committee-approved protocol (Protocol #1807-7C). Genotyping was ascertained as previously described (Vomhof-DeKrey et al., 2019).

### RNA isolation and qPCR

We isolated intestinal mucosa, thymus and spleens from male and female Slfn3KO and WT littermates that were 8–20 weeks old. Total RNA from harvested tissues was isolated using the RNeasy Lipid Kit and the QiaCube instrument per manufacturer’s protocols (Qiagen, Valencia, CA, USA). cDNA synthesis was performed using the SMARTScribe Reverse Transcription kit (Takara Clontech, Mountain View, CA, USA). qPCR analysis was done using the BioRad CFX96 Touch Real-Time PCR Detection System and the PrimeTime Gene Expression Master Mix from Integrated DNA Technology (IDT, Coralville, IA, USA). RPLP0 was used as the reference control gene and the expression levels were elucidated from the threshold cycle (Ct) values using the method of 2^−∆∆Ct^. The primer/probe sets used from BioRad (Hercules, CA, USA) are proprietary: mouse RPLP0 (Assay ID: qMmuCEP0042968, HEX), mouse Slfn3 (Assay ID: qMmuCEP0053101, FAM) and mouse Slfn4 (Assay ID: qMmuCIP0035897, Cy5). Primer/probe set sequences used from IDT were as follows: mouse Slfn1 forward 5′-CTC AGT AGA GCA GCT TAG GT-3′, reverse 5′-GAT TCT CCA TGG TTT TCC TAC TTT C-3′, probe 5′-/56-FAM/TGC ATT TAG/ZEN/AAT CAG CAC AGG GGT CC/3IABkFQ/-3′; mouse Slfn2 forward 5′-AAG CAA CCA CAG AAC TCA GA-3′, reverse 5′-CCG AGA TTT AGA CCC AGC TTA G-3′, probe 5′-/5HEX/AAC ACT GAT/ZEN/GCC CAT TTT CCC AGC/3IABkFQ/-3′; mouse Slfn5 forward 5′-CAG CAA GAC CGT GTT CAC A-3′, reverse 5′-CTT AGT CCT CTG TAA TCT GCG AA-3′; probe 5′-/5Cy5/TCC TTC TGC AGG TCT TCC ACA CAT TT/3IAbRQSp/-3′; mouse Slfn8 forward 5′-ACT GTG TCT CTC CTG TCT GA-3′, reverse 5′-GGA TGT GTC TCC ATG CTG AT-3′, probe 5′-/56-FAM/CCC GGA TAT/ZEN/TTT CTC AGA CTT GGC GA/3IABkFQ/-3′; and mouse Slfn9 forward 5′-ACT GGA ACA TAA AGT CGA CCT G-3′, reverse 5′-CTA AAT CCT CTA GTC TCT CAT GCT G-3′, probe 5′-/5Cy5/CCT GAA TCT CTC TGG AGT GAG CTG TGT/3IAbRQSp/-3′. qPCR cycle conditions were 1 cycle of 2 min at 95 °C, 50 cycles of 10 s at 95 °C and 45 s at the annealing temperature of 55 °C, as previously described (Vomhof-DeKrey et al., 2019).

### RNA sequencing

From four male and female WT and Slfn3KO mice, intestinal ileum mucosa was harvested, and RNA was isolated as stated above. RNA sequencing methods were previously described but are briefly as follows (Vomhof-DeKrey et al., 2019). RNA quality and concentration was analyzed on a Qubit 2.0 Fluorometer (ThermoFisher Scientific, Waltham, MA, USA) by the UND Genomics Core. The Illumina HiSeq 2500 was utilized to perform RNA sequencing with a high output mode, single read, 50 bp and 2 lanes were run for each sample. Data was analyzed by Rosalind (https://rosalind.onramp.bio/), with a HyperScale architecture developed by OnRamp BioInformatics, Inc. (San Diego, CA, USA). Reads were trimmed using cutadapt (Martin, 2011). Quality scores were assessed using FastQC (Andrews, 2014). Reads were aligned to the *Mus musculus* genome build mm10 using STAR (Dobin et al., 2013). Individual sample reads were quantified using HTseq (Anders, Pyl & Huber, 2015) and normalized via Relative Log Expression (RLE) using DESeq2 R library (Love, Huber & Anders, 2014). DEseq2 was also used to calculate fold changes and *p*-values. Clustering of genes for differentially expressed genes was done using the Partitioning Around Medoids method using the fpc R library (https://cran.r-project.org/web/packages/fpc/index.html). The RNA sequencing data has been uploaded to Gene Expression Omnibus and the accession number is GSE132268. Code used to generate the simulated data can be found at https://www.ncbi.nlm.nih.gov/geo/query/acc.cgi?acc=GSE132268.

### Statistics

The differential expression RNA sequencing analysis of all Slfn3KO samples vs. WT samples were obtained using a threshold of 0.05 for statistical significance (*p*-value) and a log fold change of expression with absolute value of at least 0.584962500721156. qPCR data was assessed by unpaired Student’s *t* test with Welch’s correction for unequal variances in GraphPad Prism (San Diego, CA, USA). Data are represented as mean ± SE.

## Results

### Loss of Slfn3 expression decreases Slfn4, Slfn8 and Slfn9 and increases Slfn1 and Slfn5 in ileal mucosa

**Figure 1:**
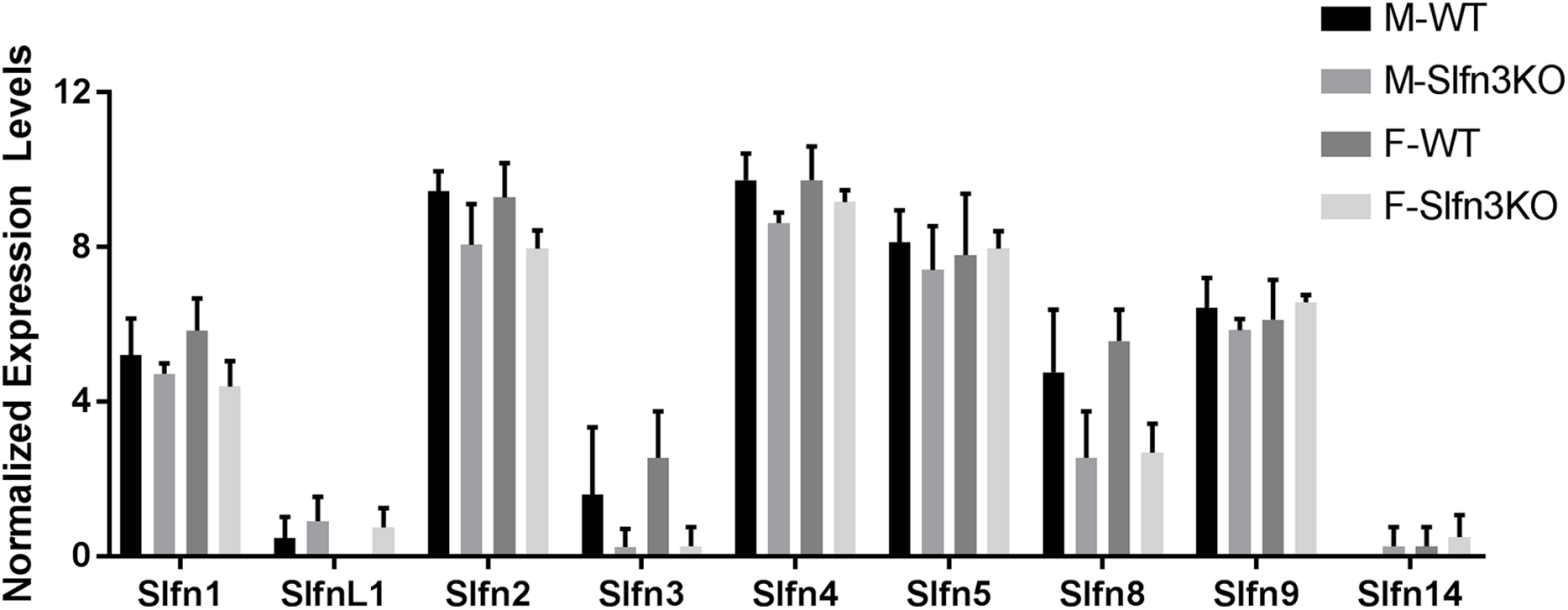
RNA sequencing expression of Slfn family members. Total RNA was isolated from intestinal mucosa of WT and Slfn3KO mice and analyzed on an Illumina HiSeq 2500 (*n* = 4 per group).

**Figure 2:**
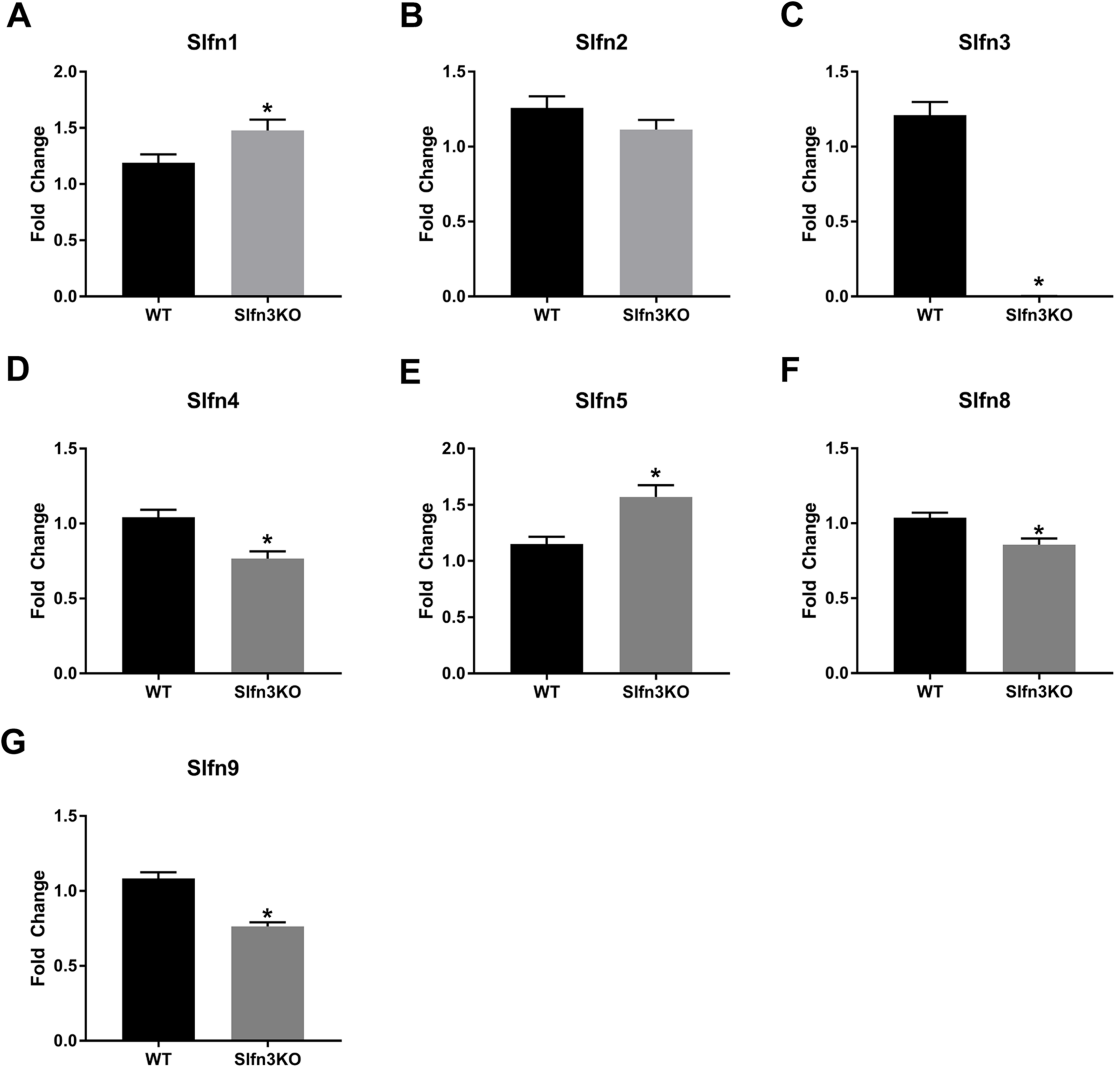
Slfn family member expression in ileum intestinal mucosa. Total RNA was isolated from intestinal mucosa of WT and Slfn3KO mice and Slfn expression was analyzed by qPCR using RPLP0 as a reference control gene. mRNA expression of (A) Slfn1, (B) Slfn2, (C) Slfn3, (D) Slfn4, (E) Slfn5, (F) Slfn8 and (G) Slfn9 (*n* = 102–115; *p < 0.05 to WT).

RNA sequencing of intestinal ileum mucosa was performed on Slfn3KO male and female mice utilizing the Illumina HiSeq 2500. From the RNA sequencing differential expression analysis, we found that the loss of Slfn3 impacted the mRNA expressions of the other Slfn family members (Fig. 1; Table 1). The RNA sequencing data is from 4 mice per group and we have previously found that an increased number of independent biological replicates of WT and Slfn3KO mice is necessary to confirm the biological conclusions indicated by the RNA sequencing data (Fang & Cui, 2011; Vomhof-DeKrey et al., 2019). Therefore, we validated the RNA sequencing data by qPCR. By qPCR, we determined a decrease in the mRNA expressions of Slfn3, 4, 8 and 9 (Figs. 2C, 2D, 2F and 2G) and an increase in the mRNA expression of Slfn1 and Slfn5 (Figs. 2A and 2E) in the ileal mucosa. There was no significant difference seen for Slfn2 (Fig. 2B). Additionally, there was no difference in Slfn mRNA expression due to sex after qPCR validation, so the qPCR data was pooled.

**Table 1:** Slfn family members that had significant differential expression changes between WT and Slfn3KO mice. DEseq2 was used to calculate fold changes and *p*-values.

Differential expression analysis	Gene	RNA seq fold change	*p*-Value
M-WT vs. M-KO	Slfn2	−1.60355	0.00436
	Slfn4	−1.77139	0.00023
F-WT vs. F-KO	Slfn2	−2.12762	0.03218
	Slfn8	−3.78247	8.74 × 10^−5^
All WT vs. All KO	Slfn1	−1.70485	0.00147
	Slfn2	−1.95915	1.83 × 10^−5^
	Slfn4	−1.66425	0.00063
	Slfn8	−2.59188	6.17 × 10^−8^

When the validated qPCR data was compared to the RNA sequencing data (Table 1; Fig. 1 vs. Fig. 2), the key differences were that Slfn1 was significantly increased in the Slfn3KO qPCR data, while the RNA sequencing data suggested a decrease for Slfn1. RNA sequencing Slfn4 and Slfn8 decrease in Slfn3KO mice was validated by qPCR. Lastly, RNA sequencing did not significantly show differences in Slfn5 and Slfn9, however qPCR validation demonstrated an increase and decrease, respectively, in the Slfn3KO mice in comparison to the WT mice. These differences seen between the RNA sequencing and the qPCR data point to the necessity of RNA sequencing validation where fold changes estimated by RNA sequencing data are from few biological replicates. Therefore, when the biological replicates are increased with qPCR analysis, a statistically significant biological conclusion can be obtained, and the variances seen with few biological replicates are then negated. Additionally our data follow reports by the Sequencing Quality Control consortium and others that have demonstrated that >80% of genes are consistent between RNA sequencing and qPCR validation, however genes that are smaller, have fewer, exons and that are more lowly expressed tend to reveal inconsistency expression between RNA sequencing and qPCR validation (Everaert et al., 2017; SEQC/MAQC-III, 2014).

### Slfn3KO thymic and splenic tissue display converse expressions of Slfn8 and 9 in comparison to ileal mucosa

**Figure 3:**
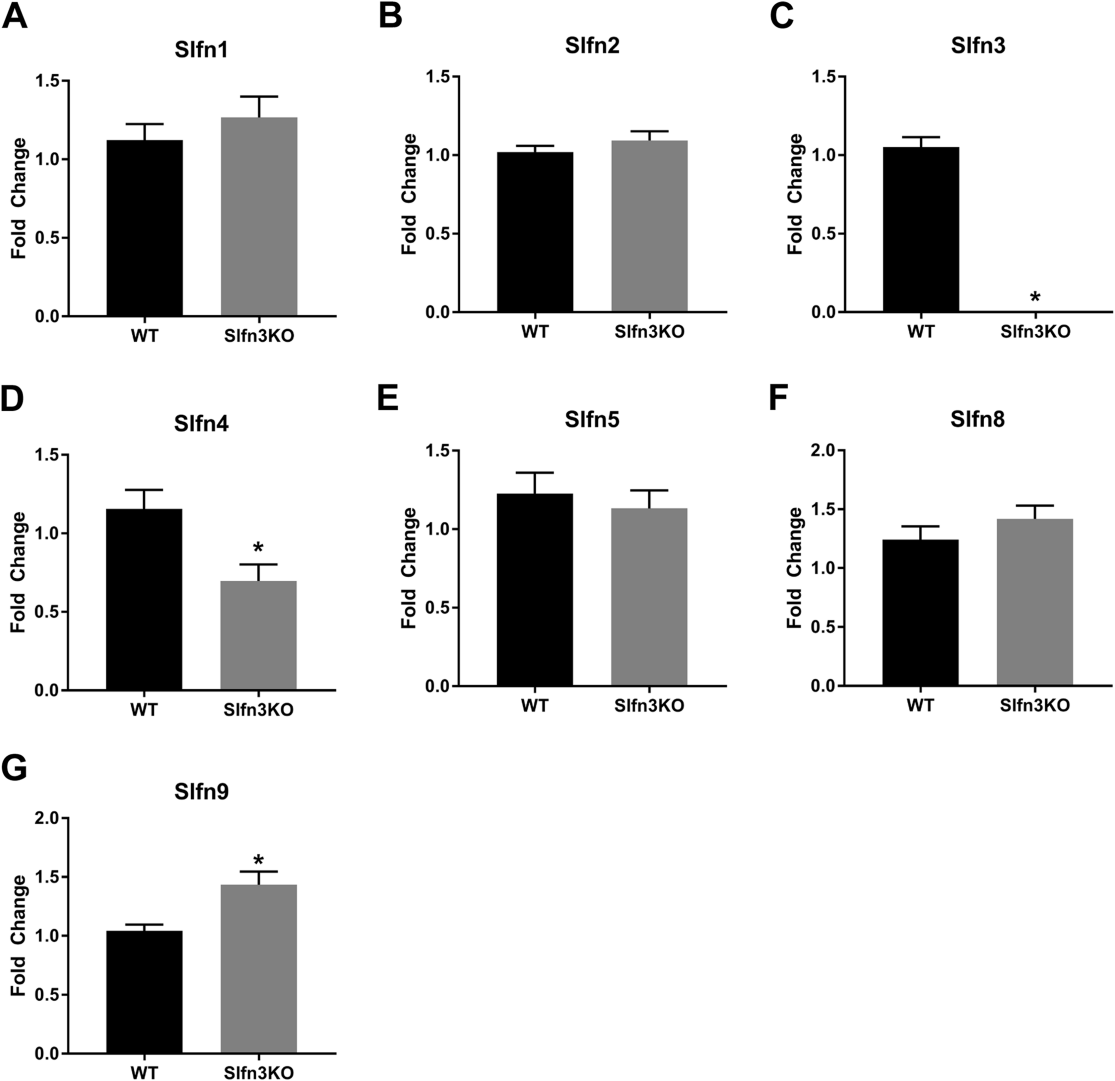
Slfn family member expression in thymus. Total RNA was isolated from thymic tissue of WT and Slfn3KO mice and Slfn expression was analyzed by qPCR using RPLP0 as a reference control gene. mRNA expression of (A) Slfn1, (B) Slfn2, (C) Slfn3, (D) Slfn4, (E) Slfn5, (F) Slfn8 and (G) Slfn9 (*n* = 23–26; *p < 0.05 to WT).

**Figure 4:**
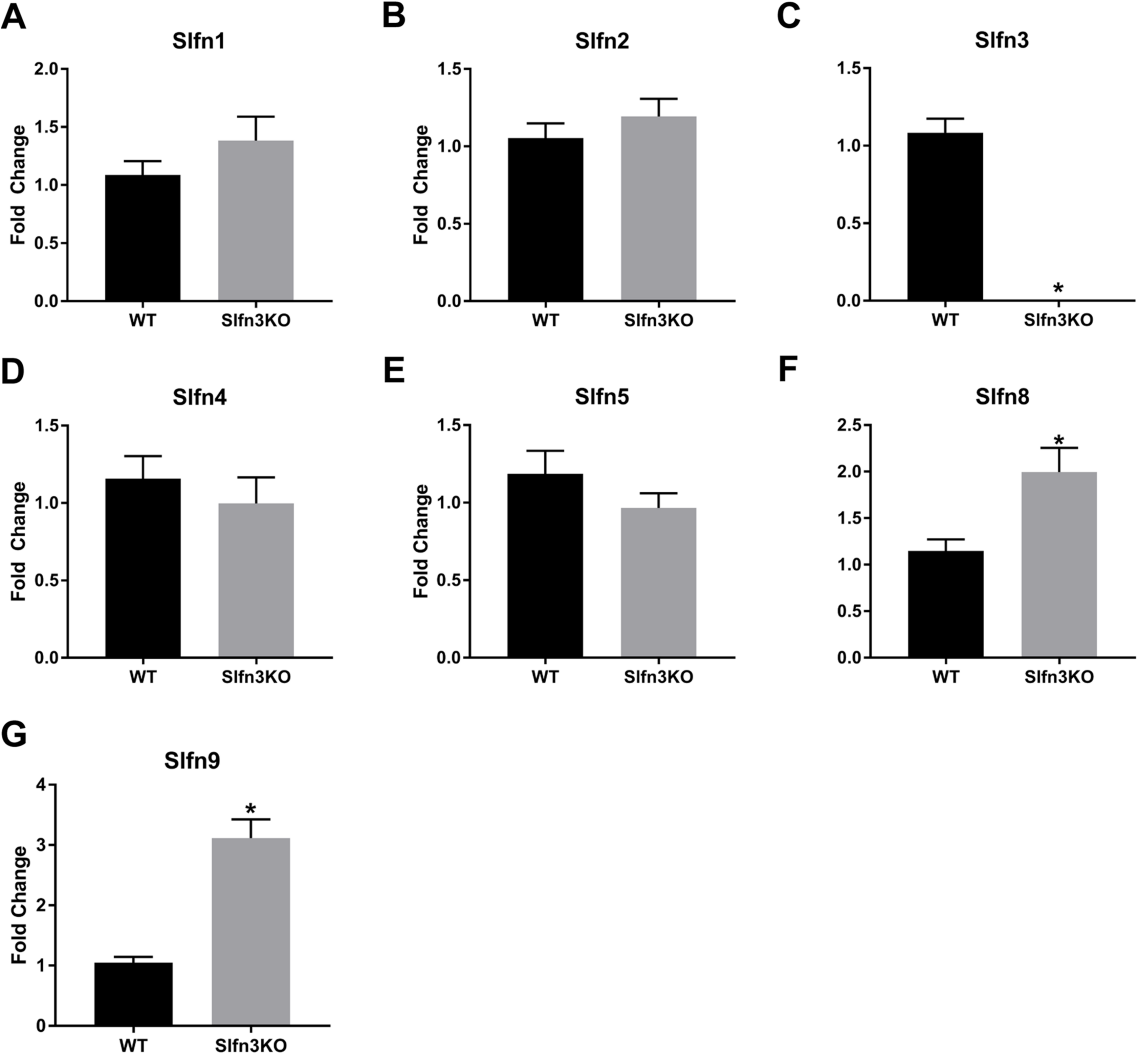
Slfn family member expression in spleen. Total RNA was isolated from splenic tissue of WT and Slfn3KO mice and Slfn expression was analyzed by qPCR using RPLP0 as a reference control gene. mRNA expression of (A) Slfn1, (B) Slfn2, (C) Slfn3, (D) Slfn4, (E) Slfn5, (F) Slfn8 and (G) Slfn9 (*n* = 22–24; *p < 0.05 to WT).

Since the intestinal mucosa is a heterogeneous population of intestinal endocytes and immune cell populations, we further explored the expression of Slfn family members in thymic and splenic tissue from Slfn3KO and WT male and female mice. Slfn3 was completely knocked out in both thymic and splenic tissues (Figs. 3C and 4C) and there were no differences between male and female mice after qPCR validation, so the qPCR data was pooled. The thymic tissue from Slfn3KO mice displayed an increase in Slfn9 (Fig. 3G). While Slfn4 was decreased in Slfn3KO mice (Fig. 3D). There were no significant changes in Slfn1, 2, 5 and 8 between the thymuses of WT and Slfn3KO mice (Figs. 3A, 3B, 3E and 3F). Slfn8 and Slfn9 were significantly increased in the Slfn3KO in comparison to the WT splenic tissue (Figs. 4F and 4G). However, there were no significant changes in Slfn1, Slfn2, Slfn4 and Slfn5 between the spleens of Slfn3KO and WT mice (Figs. 4A, 4B, 4D and 4E).

## Discussion

The regulation of Slfn family member expression is poorly understood. This study suggests that the loss of Slfn3 differentially effects the expression of other Slfn family members. Furthermore, we observed differential changes in RNA expression of other Slfn family members among the thymus, spleen and intestinal mucosa tissue compartments which has been summarized in Table 2. It would have been interesting to immunoblot for these various Slfn family members to further extend our mRNA data. However, this must await the availability of reliable Slfn-family member-specific antibodies for this purpose. The Slfn family members were most effected by the loss of Slfn3 in the ileal mucosa (Table 2). There was a converse expression of Slfn8 and Slfn9 with a decreased expression in the ileal mucosa but an increased expression in the splenic tissue for both Slfn8 and Slfn9, and an increased expression in Slfn9 only in thymic tissue (Table 2).

**Table 2:** Summary of Slfn family RNA expression changes in Slfn3KO vs. WT mice. Arrows indicate an increase (↑) or decrease (↓) in Slfn family expression of Slfn3KO mice vs. WT mice. N.D. indicates no difference between Slfn3KO and WT mice.

	Ileum	Thymus	Spleen
Slfn1	↑	N.D.	N.D.
Slfn2	N.D.	N.D.	N.D.
Slfn3	↓	↓	↓
Slfn4	↓	↓	N.D.
Slfn5	↑	N.D.	N.D.
Slfn8	↓	N.D.	↑
Slfn9	↓	↑	↑

Regulation of Slfn family expression has yet to be fully described. In intestinal cells, Slfn3 expression or the human homolog, Slfn12, increases with the use of differentiating agents, butyrate (Basson et al., 2000; Chaturvedi et al., 2019; Emenaker & Basson, 1998; Kruh, 1982; Langdon et al., 1988; Ren et al., 2010), TGF-β (Heldin, Landstrom & Moustakas, 2009; Romano, 2009; Watabe & Miyazono, 2009; Yuan et al., 2010) and cyclic strain (Chaturvedi et al., 2019; Yuan et al., 2010). Slfn2 inhibits malignant cell proliferation after stimulation through the Type I interferon receptor, Stat- and p38-MAPK pathways, and suppression of cyclin D1 (Katsoulidis et al., 2009). While mouse Slfn1–5, Slfn8–9 and Slfn14 and human Slfn5 and Slfn11–14 are all induced by Type I interferon-stimulated genes such as Jak kinases- phosphatidylinositol-3- kinase, mammalian target of rapamycin, mitogen-activated protein kinases (MAPK), and signal transducer and activator of transcription (STAT) (Mavrommatis, Fish & Platanias, 2013).

The differential expression of Slfn family members in response to the loss of Slfn3 could indicate a possible feedback regulatory mechanism within the Slfn family. Utilizing the MATCH^™^ tool from geneXplain (Mavrommatis, Fish & Platanias, 2013), we found that all Slfn family members have regions in their promoters for the binding of Kruppel-like factor-6 (KLF6) transcription factor (Table 3; Excel File S1). Additionally, NFAT related factors, ING4, ZNF333 and KLF4 are predicted to bind in up to 6 of the 8 Slfn promoters (Table 2; Excel File S1). KLF4 is expressed in the terminally differentiated villus cells while KLF5 is expressed in the proliferating crypt cells (Flandez et al., 2008). KLF6 interacts with KLF4 in order to co-activate keratin-4 which associates with the proliferation to differentiation switch in human esophageal squamous cancer cells (Ghaleb & Yang, 2008; Okano et al., 2000). Such observations would be consistent with a model in which there is a regulatory feedback loop between the KLF family and Slfn family members as they have all been recognized as regulators of gastrointestinal and immune cell differentiation (Bieker, 2001; Flandez et al., 2008; Ghaleb & Yang, 2008; Liu et al., 2018; Mavrommatis, Fish & Platanias, 2013; Okano et al., 2000). However, this awaits further exploration beyond the scope of the current manuscript.

**Table 3:** Transcription factors with predicted binding in the promoter regions of Slfn family members. Shading indicates similar transcription factors binding within 2 or more Slfn family members.

Slfn1	Slfn2	Slfn3	Slfn4	Slfn5	Slfn8	Slfn9
KLF6	KLF6	KLF6	KLF6	KLF6	KLF6	KLF6
NFAT-related factors	NFAT-related factors		NFAT-related factors	NFAT-related factors	NFAT-related factors	NFAT-related factors
ING4	ING4	ING4			ING4	ING4
ZNF333		ZNF333	ZNF333	ZNF333	ZNF333	
KLF4 group	KLF4 group	KLF4 group		KLF4 group		KLF4 group
AP-2	AP-2	AP-2				AP-2
	Tef-1-related factors			Tef-1-related factors	Tef-1-related factors	Tef-1-related factors
		Sox-related factors	Sox-related factors	Sox-related factors		Sox-related factors
NF-kappaB-related factor	NF-kappaB-related factor			NF-kappaB-related factor		
TBX5	TBX5	TBX5			TBX5	
Myogenin group		Myogenin group		Myogenin group	Myogenin group	
Ikaros		Ikaros	Ikaros			
			BEN	BEN	BEN	BEN
RFX-related factors					RFX-related factors	RFX-related factors
DRI1				DRI1	DRI1	
HMGA factors			HMGA factors	HMGA factors		
	Paired related HD factors	Paired related HD factors	Paired related HD factors			
	MAF group	MAF group			MAF group	
	Blimp-1		Blimp-1	Blimp-1		
		Myb-like factors		Myb-like factors		Myb-like factors
		CDX group	CDX group (half-site)	CDX group		
BRCA1		BRCA1				
Ebox						Ebox
SREBP factors			SREBP factors			
	AP-1			AP-1		
	Bbx					Bbx
	GATA					GATA
	FOXC		FOXC			
	REST			REST		
	MAZ		MAZ			
	MZF1			MZF1		
	DMRT	DMRT				
	RREB-1			RREB-1		
		GFI1 factors	GFI1 factors			
			YY1-like factors	YY1-like factors		
				ATF-2 group	ATF-2 group	
POU1F1	POU domain group	FAC1	POU domain group	FPM315	POU6F1	HSF dimer
Sox10	MECP2	ER group (half-site)	AhR	NR-DR	BCL-6 factors	
TTF-1 (Nkx2.1)	C/EBP group	CDC5L	Xvent-1	Sp100	Runt-related factors	
p53 related factors	Nkx group	TBP-related factors	TBX5	Early B-Cell Factor-related factors	E2A group	
GR-like receptors		E2F related factors	CP2-related factors	Sp1 group	CDP	
				Churchill	LRH-1 group	
				HNF1-like factors	SF-1 group	

To date, several authors have investigated the role of Slfn1, Slfn2, Slfn3, Slfn4 and Slfn8, in immune cell development and function. Slfn1 or Slfn8 thymic- or myeloid-overexpressing transgenic specific mice have a reduction in thymic cellularity and impaired T cell development (Geserick et al., 2004; Schwarz, Katayama & Hedrick, 1998). Slfn2 knockout (*elektra*) mice are more susceptible to bacterial and viral infections and have diminished T cell and monocyte numbers (Berger et al., 2010). Slfn3 has been shown to be downregulated in CD4^+^ CD25^+^ T regulatory cells and upregulated in CD4^+^ CD25^−^ T effector cells, therefore indicating a role for Slfn3 in T cell differentiation and activation (Condamine et al., 2010). Slfn4 is involved in differentiation of the monocyte/macrophage lineage and macrophage activation (De La Casa-Esperon, 2011; Van Zuylen et al., 2011). Additionally, several inflammatory diseases and infections are accompanied by changes to multiple Slfn genes differential expressions (De La Casa-Esperon, 2011). The macrophage-mediated disease, rheumatoid arthritis is associated with a region containing the Slfn gene cluster (Fujikado, Saijo & Iwakura, 2006) and the mouse model for rheumatoid arthritis has increased expressions of Slfn1, Slfn2 and Slfn4 in the joints (Van Zuylen et al., 2011). The expression levels of Slfn1, Slfn2 and Slfn3 are higher in the lung of pneumonia-resistant C57BL/6 strain of mice after a low-dose infection with *Klebsiella pneumoniae* (Schurr et al., 2005). Even with all of these data demonstrating roles of Slfn family members in the regulation of immune cell development and function, little is known about the effects of the Slfn family members on each other and whether this is contributing to the alterations of cell differentiation and function within the immune system.

It is interesting that the levels of Slfn8 and Slfn9 mRNA were oppositely affected by the loss of Slfn3 in the ileal mucosa and spleen tissue compartments. One possible reason for differential expression levels of Slfn8 and Slfn9 in the intestinal mucosa vs. splenic tissue of the Slfn3KO mice compared to the WT mice, is the overall makeup of the tissue environment and the difference in the immune cell roles in the intestinal mucosa as opposed to the spleen. Specifically, murine Slfn8 has been shown to be downregulated during activation of T cells while Slfn9 is induced (Geserick et al., 2004; Schwarz, Katayama & Hedrick, 1998). Therefore, depending upon activation states of the immune cells in the intestinal mucosa and the spleen, the expression levels of Slfn8 and Slfn9 would be expected to fluctuate.

It is intriguing that the loss of Slfn3 would affect the RNA expression levels of Slfn8 and Slfn9 especially since Slfn8 and 9 are localized to the nucleus while Slfn3 is in the cytoplasm. However, transfected Slfn3 in the cytosol mediates human Caco2 intestinal epithelial cell differentiation by triggering a secondary signal cascade via the Slfn3 P-Loop active domain (Chaturvedi et al., 2014). Indeed, this occurs even when the entry of the over expressed Slfn3 into the nucleus is prevented by adding a nuclear exclusion sequence (Chaturvedi et al., 2014). Cytosolic SLFN12, which is the likely human ortholog of Slfn3, regulates human enterocytic differentiation via a pathway involving SerpinB12 and the deubiquitylases USP14 and UCHL5 to change expression of the transcription factor Cdx2 which in turn modulates human gene expression (Basson et al., 2018). Such cytosolic modulation of gene expression Slfn3 could modulate the expression of other Slfn family members through such pathways.

## Conclusions

In this paper, we report that the loss of Slfn3 results in mRNA expression changes of Slfn family members in the intestinal mucosa, thymus and spleen. These data offer insight into Slfn family member regulation in which not much is known. These data show that there could be a regulatory feedback loop between Slfn members in both intestinal and immune cell types. Overall, with the Slfn family being highly involved with the regulation and differentiation of various cell types, understanding how Slfns themselves are influenced will promote the development of new treatments for cancer and immune diseases and infections.

## Supplemental Information

10.7717/peerj.8461/supp-1 Supplemental Information 1 Transcription factor binding to Slfn family promoters.

10.7717/peerj.8461/supp-2 Supplemental Information 2 Raw qPCR data plate 2 for Slfn family in the spleen utilized for data analyses and preparation of graph shown in Fig. 4.

10.7717/peerj.8461/supp-3 Supplemental Information 3 Raw qPCR data plate 5 for Slfn family in the ileal mucosa or spleen or thymus utilized for data analyses and preparation of graphs shown in Fig. 2, 3 and 4.

10.7717/peerj.8461/supp-4 Supplemental Information 4 Raw qPCR data plate 4 for Slfn family in the spleen or thymus utilized for data analyses and preparation of graphs shown in Fig. 3 and 4.

10.7717/peerj.8461/supp-5 Supplemental Information 5 Raw qPCR data plate 1 for Slfn family in the thymus utilized for data analyses and preparation of graph shown in Fig. 3.

10.7717/peerj.8461/supp-6 Supplemental Information 6 Raw qPCR data plate 3 for Slfn family in the ileal mucosa utilized for data analyses and preparation of graph shown in Fig. 2.

10.7717/peerj.8461/supp-7 Supplemental Information 7 Raw qPCR data plate 1b for Slfn family in the thymus utilized for data analyses and preparation of graphs shown in Fig. 3.

10.7717/peerj.8461/supp-8 Supplemental Information 8 Raw qPCR data plate 1c for Slfn family in the thymus utilized for data analyses and preparation of graphs shown in Fig. 3.

10.7717/peerj.8461/supp-9 Supplemental Information 9 Raw qPCR data plate 4 for Slfn family in the spleen utilized for data analyses and preparation of graphs shown in Fig. 4.

10.7717/peerj.8461/supp-10 Supplemental Information 10 Raw qPCR data plate 3b for Slfn family in the spleen or thymus utilized for data analyses and preparation of graphs shown in Fig. 3 and 4.

10.7717/peerj.8461/supp-11 Supplemental Information 11 Raw qPCR data plate 5b for Slfn family in the intestinal mucosa or spleen or thymus utilized for data analyses and preparation of graphs shown in Fig. 2, 3 and 4.

10.7717/peerj.8461/supp-12 Supplemental Information 12 Raw qPCR data plate 7 for Slfn family in the ileal mucosa utilized for data analyses and preparation of graphs shown in Fig. 2.

10.7717/peerj.8461/supp-13 Supplemental Information 13 Raw qPCR data plate 2 for Slfn family in the thymus utilized for data analyses and preparation of graphs shown in Fig. 3.

10.7717/peerj.8461/supp-14 Supplemental Information 14 Raw qPCR data plate 4 for Slfn family in the spleen utilized for data analyses and preparation of graphs shown in Fig. 4.

10.7717/peerj.8461/supp-15 Supplemental Information 15 Raw qPCR data plate 8 for Slfn family in the ileal mucosa utilized for data analyses and preparation of graphs shown in Fig. 2.

10.7717/peerj.8461/supp-16 Supplemental Information 16 Raw qPCR data plate 6 for Slfn family in the ileal mucosa utilized for data analyses and preparation of graphs shown in Fig. 2.

10.7717/peerj.8461/supp-17 Supplemental Information 17 Raw qPCR data plate 6b for Slfn family in the ileal mucosa utilized for data analyses and preparation of graphs shown in Fig. 2.

10.7717/peerj.8461/supp-18 Supplemental Information 18 Raw qPCR data of Slfn family multiplexing test plate to determine primer/probe sets that work well together for further analyses.

10.7717/peerj.8461/supp-19 Supplemental Information 19 Raw qPCR data plate 1 for Slfn family in the ileal mucosa utilized for data analyses and preparation of graphs shown in Fig. 2.

10.7717/peerj.8461/supp-20 Supplemental Information 20 Raw qPCR data plate 3 for Slfn family in the ileal mucosa utilized for data analyses and preparation of graphs shown in Fig. 2.

10.7717/peerj.8461/supp-21 Supplemental Information 21 Raw qPCR data plate 9 for Slfn family in the ileal mucosa utilized for data analyses and preparation of graphs shown in Fig. 2.

10.7717/peerj.8461/supp-22 Supplemental Information 22 Raw qPCR data plate 10 for Slfn family in the ileal mucosa utilized for data analyses and preparation of graphs shown in Fig. 2.

10.7717/peerj.8461/supp-23 Supplemental Information 23 Raw qPCR data plate 12 for Slfn family in the ileal mucosa utilized for data analyses and preparation of graphs shown in Fig. 2.

10.7717/peerj.8461/supp-24 Supplemental Information 24 Raw qPCR data plate 11 for Slfn family in the ileal mucosa utilized for data analyses and preparation of graphs shown in Fig. 2.

10.7717/peerj.8461/supp-25 Supplemental Information 25 Raw qPCR data plate 2 for Slfn family in the ileal mucosa utilized for data analyses and preparation of graphs shown in Fig. 2.

10.7717/peerj.8461/supp-26 Supplemental Information 26 Raw qPCR data plate 5 for Slfn family in the ileal mucosa utilized for data analyses and preparation of graphs shown in Fig. 2.

10.7717/peerj.8461/supp-27 Supplemental Information 27 Raw qPCR data plate 6 for Slfn family in the ileal mucosa utilized for data analyses and preparation of graphs shown in Fig. 2.

10.7717/peerj.8461/supp-28 Supplemental Information 28 Raw qPCR data plate 4 for Slfn family in the ileal mucosa utilized for data analyses and preparation of graphs shown in Fig. 2.

10.7717/peerj.8461/supp-29 Supplemental Information 29 Raw qPCR data plate 7 for Slfn family in the ileal mucosa utilized for data analyses and preparation of graphs shown in Fig. 2.

10.7717/peerj.8461/supp-30 Supplemental Information 30 Raw qPCR data plate 8 for Slfn family in the ileal mucosa utilized for data analyses and preparation of graphs shown in Fig. 2.

## Additional Information and Declarations

### Data Availability

The following information was supplied regarding data availability: The RNA sequencing data and code used to generate the simulated data are available at the Gene Expression Omnibus: GSE132268.
